# Different gene-expression profiles for the poorly differentiated carcinoma and the highly differentiated papillary adenocarcinoma in mammary glands support distinct metabolic pathways

**DOI:** 10.1186/1471-2407-8-270

**Published:** 2008-09-24

**Authors:** Tali Eilon, Itamar Barash

**Affiliations:** 1 Institute of Animal Science, ARO, The Volcani Center, Bet-Dagan, Israel

## Abstract

**Background:**

Deregulation of Stat5 in the mammary gland of transgenic mice causes tumorigenesis. Poorly differentiated carcinoma and highly differentiated papillary adenocarcinoma tumors evolve. To distinguish the genes and elucidate the cellular processes and metabolic pathways utilized to preserve these phenotypes, gene-expression profiles were analyzed.

**Methods:**

Mammary tumors were excised from transgenic mice carrying a constitutively active variant of Stat5, or a Stat5 variant lacking s transactivation domain. These tumors displayed either the carcinoma or the papillary adenocarcinoma phenotypes. cRNAs, prepared from each tumor were hybridized to an Affymetrix GeneChip^® ^Mouse Genome 430A 2.0 array. Gene-ontology analysis, hierarchical clustering and biological-pathway analysis were performed to distinct the two types of tumors. Histopathology and immunofluorescence staining complemented the comparison between the tumor phenotypes.

**Results:**

The nucleus-cytoskeleton-plasma membrane axis is a major target for differential gene expression between phenotypes. In the carcinoma, stronger expression of genes coding for specific integrins, cytoskeletal proteins and calcium-binding proteins highlight cell-adhesion and motility features of the tumor cells. This is supported by the higher expression of genes involved in *O*-glycan synthesis, TGF-β, activin, their receptors and Smad3, as well as the Notch ligands and members of the γ-secretase complex that enable Notch nuclear localization. The Wnt pathway was also a target for differential gene expression. Higher expression of genes encoding the degradation complex of the canonical pathway and limited TCF expression in the papillary adenocarcinoma result in membranal accumulation of β-catenin, in contrast to its nuclear translocation in the carcinoma. Genes involved in cell-cycle arrest at G1 and response to DNA damage were more highly expressed in the papillary adenocarcinomas, as opposed to favored G2/M regulation in the carcinoma tumors.

**Conclusion:**

At least six metabolic pathways support the morphological and functional differences between carcinomas and papillary adenocarcinomas. Differential gene-expression profiles favor cell adhesion, motility and proliferation in the carcinoma. Cell-cell contact, polarity, earlier cell-cycle arrest and DNA damage control are better displayed in the papillary adenocarcinoma.

## Background

Breast cancer comprises a series of distinct malignant tumors that present diverse cellular features with different stages and grades, distinct genetic changes, differing responses to therapy and varying outcomes [1]. Tailoring specific treatments to the disease's subtypes has traditionally been performed by histopathological analysis of tumor sections, supported by limited immunopathological and genetic assays [2]. Gene-expression profiling of human breast cancers has expanded our understanding of the clinical diversity of the disease and enabled a more accurate classification of tumors into subtypes, as well as a determination of their response to drug treatments [3,4]. The clinical benefits gained from profiling gene expression in tumor biopsies have also provided better insight into the development and characteristics of the disease. For instance, the discovery of a unique set of genes that are predictive of metastases was associated with the recognition that metastatic properties are determined in the primary tumor relatively early in development. It also indicated that the molecular mechanism involved in bone marrow metastasis is different from that mediating lymphatic spread [5-8]. Likewise, the "proliferation signature" encompasses a universal pattern of gene expression among tissues and predicts the outcome in patients [9]. It also implies that the regulation of some individual cell-cycle regulatory genes is more complex than simple restriction of transcription to certain phases of the cell cycle.

Indeed, progress has been made in determining the molecular profiles of breast-tumor subtypes and their response to treatment [5,6,10,11]. However, the distinct contribution of defined metabolic pathways and their primary elements to the overall phenotypic and functional diversity among tumors remains poorly understood.

In breast cancer, rate of differentiation has been negatively correlated with the invasiveness and aggressiveness of the disease [12]. To elucidate the molecular and cellular characteristics and putative pathways that distinguish the poorly differentiated carcinoma and the highly differentiated papillary adenocarcinoma, global gene expression analysis was performed in transgenic mouse model. The tumors develop in about 8 to 9% of the aged, post-estropausal transgenic mice expressing variants of the signal transducer and activator of transcription 5 (Stat5) in their mammary glands. Both tumor types are of epithelial origin, but strictly different in their pathological appearance. The carcinoma is an undifferentiated, non-glandular tumor, which can be distinguished by a typical structure of sheets of neoplastic mass with solid nests of poorly organized and discohesive cells. In contrast, the papillary adenocarcinoma is a highly differentiated tumor of glandular origin. Its microscopic structure is composed predominantly of fibrovascular frond-like projections, covered by epithelial cells [13,14]. Divergent protein expression which is not associated with the type of transgenic Stat5 variant expressed has been defined in these tumors, and a high degree of aneuploidy was demonstrated exclusively for the carcinoma epithelial cells [13].

Typically, cancer cells overexpress genes that are preferentially expressed in tissues other than those of the cancer's origin [15]. Thus, the apparent differences between these types of tumors could involve alterations in cellular and molecular functions, some of which are difficult to predict. Global profiling of gene expression in these tumors has revealed the nucleus-cytoskeleton-plasma membrane axis as a target for altered gene expression which determines the diversity among these tumors.

## Methods

### Mouse mammary-tumor samples

Mammary tumors were developed in transgenic mice carrying one of two Stat5 variants on a FVB/N background: (i) constitutively activated STAT5, termed STAT5ca, comprising sequences from three genes: amino acids 1–750 from ovine Stat5, 677–847 from human Stat6, and 757–1129 from mouse Janus kinase (Jak) 2. and (ii) a deleted construct, STAT5Δ750, prepared by introducing a stop codon at the respective site of the native Stat5 DNA sequence, thus eliminating the expression of its transactivation domain. These constructs were inserted into the β-lactoglobulin (BLG) multiple-cloning site for mammary-gland-specific expression [16]. Upon identification, tumors were excised and snap-frozen for RNA isolation and validation studies. Biopsies were taken for histological analyses.

All animals used in this study received humane care. The study protocols are in compliance with the regulation of the Israeli Ministry of Health and the local institutional policies (approval no. IL-39-03).

### Microarray hybridization and data analysis

RNA was extracted from the individual tumors that were pathologically diagnosed [13], or from mammary glands with TRIZOL and reverse-transcribed. Each tumor or mammary gland was excised from different mouse. Equal amounts of cRNA from each tumor were hybridized to an Affymetrix GeneChip^® ^Mouse Genome 430A 2.0 array (Affymetrix, Santa Clara, CA), which includes approximately 14,000 annotated genes from the mouse genome. Hybridization and signal quantitation were performed according to Affymetrix's protocol by the Biological Services of the Weizmann Institute of Science (Rehovot, Israel). Briefly, 15 μg total RNA was reversed-transcribed using a T7-oligo(dT) promoter-primer in the first-strand DNA-synthesis reaction. Following RNase H-mediated second-strand cDNA synthesis, the double-stranded cDNA was purified and used as a template for the subsequent in-vitro transcription reaction. This reaction was carried out in the presence of T7-RNA polymerase and a biotinylated nucleotide analogue/ribonucleotide mix for complementary RNA (cRNA) amplification and biotin labeling. The biotinylated cRNA targets were then cleaned up, fragmented, and hybridized to the GeneChip expression array. The chip was reacted with streptavidin-phycoerythrin and then with biotinylated anti-streptavidin antibody (Vector Laboratories, Burlingame, CA). Arrays were scanned by GeneArray scanner G2500A (Hewlett Packard, Palo Alto, CA), visually inspected for hybridization imperfections and analyzed using Affymetrix Microarray Suite software version 5.0 by scaling to an average intensity of 250. The data were analyzed with GeneSpring (Silicon Genetics, Redwood City, CA) using the MAS5 algorithm [17].

First, values were set below the cutoff to the cut off: 0.01. Than, Gene-expression data were normalized "per chip" and "per gene". For "per chip" normalization, all expression data on a chip were normalized to the 50^th ^percentile of the measurements taken from all values on that chip. "Per gene" normalization divide each gene in the selected samples by the median of the gene's measurements in the respective control group. It was performed here according the type of comparison being made. When expression profiles were compared between carcinoma and papillary adenocarcinoma tumors, the expression of a given gene was normalized to the median of the expression level of the wild-type mammary-gland samples.

This enabled the relation of gene expression levels in the tumor types also to that detected in the wild-type gland. For comparison between tumors and wild-type mammary gland, the expression of a given gene was normalized to the median of the data obtained from the expression of all genes an all chips. The normalized data were log-transformed. The significant (p < 0.05) differences in gene expression, based on the individual values obtained from each tumor sample, between the two types of tumors or between each tumor type and the wild-type mammary gland were calculated using one-tailed t-test statistical analysis.

### Gene-ontology analysis, hierarchical clustering and biological-pathway analysis

Genes with different levels of expression between tumor phenotypes were categorized into Cellular component, Molecular function and Biological process categories using the GO Slim annotation tool in GeneSpring. Genes assembled in each functional annotation were further separated into GO Slim terms.

Hierarchical clustering was performed on genes exhibiting a twofold, significant (*P *≤ 0.05) difference in their expression between phenotypes. These genes were clustered into four groups according to their assembly in the gene tree and condition tree and were annotated by GO using DAVID software [18]. The rate of enrichment in the relevant genes was determined for each term. The genes exhibiting differential expression in mammary carcinomas vs. papillary adenocarcinomas were assembled by the DAVID software into metabolic pathways using the Kyoto Encyclopedia of Genes and Genomes (KEGG) pathway analysis with some modifications.

### Validation of array analysis

Semi-quantitative RT-PCR was applied to confirm the different expression patterns of selected genes in the different types of tumors. RNA, extracted from individual tumors and mammary glands, was reversed-transcribed and analyzed using the primers listed in Additional file 1 (data sheet A). The number of PCR cycles was calibrated for the exponential segment of the reaction. The DNA was blotted onto a nylon membrane and reacted with the relevant ^32^P-labeled probe. The membranes were exposed to film and signals were visualized and quantitated using GelPro software.

### Histopathology and immunofluorescence staining

Mammary and tumor biopsies were fixed with Bouin's solution, dehydrated in a graded series (50 to 100%) of ethanol, cleared in xylene and embedded in paraffin. Sections of 4 μm were processed for hematoxylin & eosin (H&E) staining. For immunohistochemical analysis of Cav-1, sections were treated as previously described [14] using anti-Cav-1 antibody (Cell Signaling, Danvers, MA) diluted 1:125. Signals were generated with EnVision reagent (Dako, Glostrup, Denmark) containing the HRP-labeled secondary antibody and diaminobenzidine (DAB) substrate (Vector Laboratories, Burlingame, CA). For immunofluorescence analysis of β-catenin, sections were reacted with anti-β-catenin antibody (Sigma, St. Louis, MO, diluted 1:2,000) and signals were generated with anti-rabbit IgG conjugated to FITC (Sigma) diluted 1:2,000. For double-staining of Cav-1 and SMA, sections were reacted overnight at 4°C with a mixture of mouse anti-SMA monoclonal antibody (Dako, diluted 1:200) and rabbit anti-Cav-1. Signals were generated after incubation for 1 h with a mixture of the appropriate secondary antibodies: donkey anti-rabbit IgG conjugated to Cy™3 (Jackson Immunoresearch Laboratories. W. Baltimore Pike, PA) or donkey anti-mouse IgG labeled with Alexa Fluor^® ^488 (Molecular Probes, Eugene, OR), both diluted 1:300. Nuclear staining was performed with DAPI (Qbiogen, Irvine, CA). Stained sections were mounted in Fluoromount (DBS, Pleasanton, CA) and visualized using a Leica fluorescent microscope (Vertrieb, Bensheim, Germany). The pathological analysis of the tumors was performed by Dr. Robert Cardiff (University of California, Davis) as previously described [13].

## Results

### Comparison of mammary carcinoma and papillary adenocarcinoma tumors: Gene Ontology (GO) analysis of differentially expressed genes

**Figure 1:**
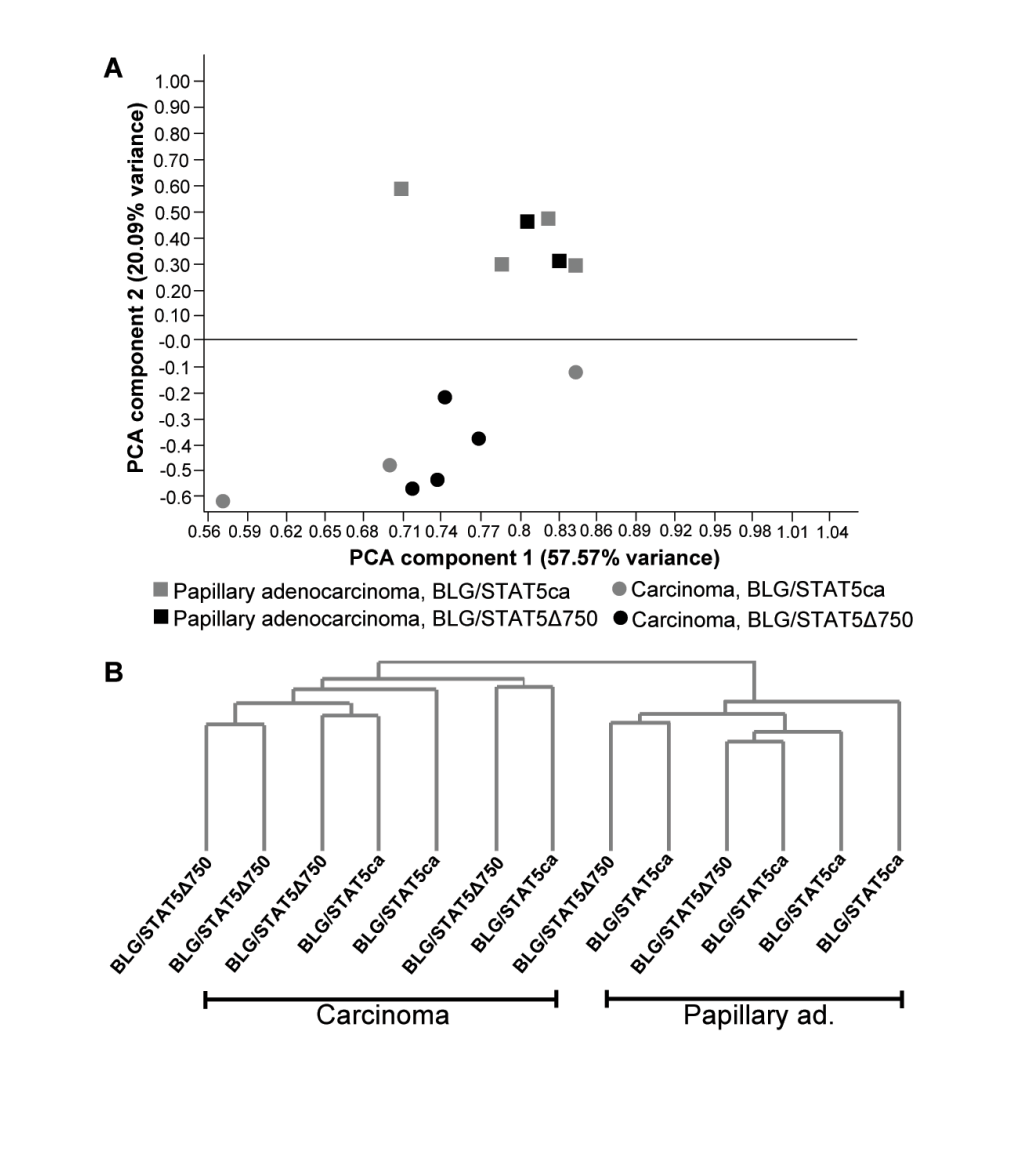
**Principal components analysis (PCA) and unsupervised hierarchical clustering into distinct mammary-tumor phenotypes.** Mammary carcinoma and adenocarcinoma tumors were developed in transgenic mice expressing the forced activated Stat5 (STAT5ca) or truncated Stat5 (STAT5Δ750). PCA (A) and unsupervised hierarchical clustering (standard correlation, B) were performed on genes that were expressed at significantly (*P *< 0.05) different levels between phenotypes and confirmed the distinction of the two phenotypes.

Global gene-expression profiling was performed on two types of mammary tumors: the poorly differentiated carcinomas and the highly differentiated papillary adenocarcinomas. RNA was extracted and reverse-transcribed from seven individual carcinoma and six individual papillary adenocarcinoma tumors that developed in transgenic mice expressing deregulated Stat5. RNA was also extracted from the mammary glands of three post-estropausal multiparous wild-type females and similarly processed. For comparison of global gene expression in the two types of tumors, the array data were normalized to the median gene-expression levels determined in the wild-type glands. This also relates the tumorigenic expression of an individual gene to its counterpart in the wild-type gland. After normalization and filtration, 2,215 features (1,882 genes) were identified that were differentially expressed in the carcinoma vs. papillary adenocarcinoma tumors at a statistical significance of *P *< 0.05 (Additional file 1, data sheet B). Principal-component analysis (PCA) and unsupervised hierarchical clustering were performed using Genespring to test the degree of similarity between the expression of the deviated genes in the two phenotypes [19]. These analyses assembled the phenotypically different genes into two sets which separated independently of the transgenic Stat5 variant carried by the host mice (Figure 1). Additional filtration of the genes to include only those exhibiting an over twofold difference narrowed the list to 865 features (773 genes; Additional file 1, data sheet C). PCA and hierarchical clustering of these genes overlapped with that obtained for the broader list of 2,215 features (not shown).

**Figure 2:**
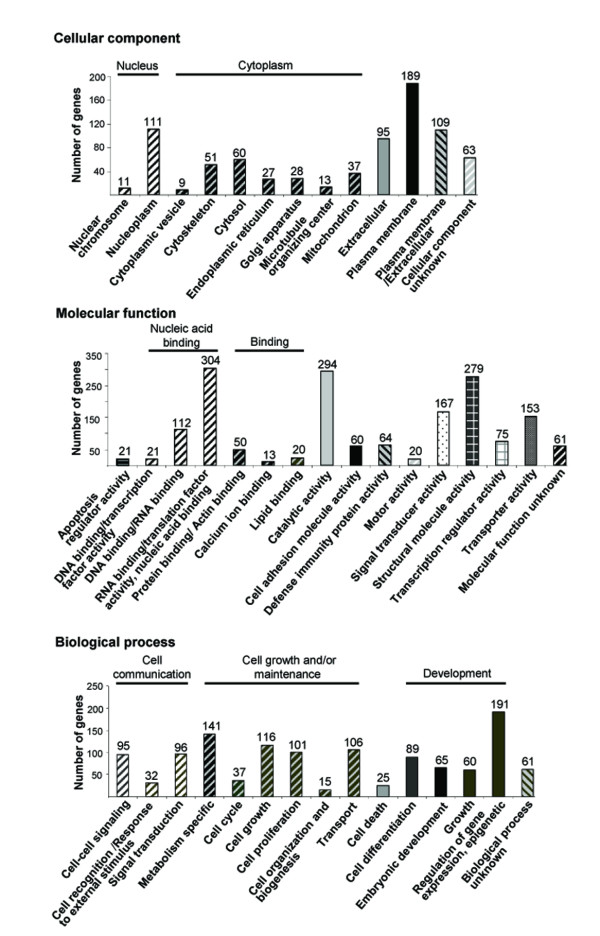
**Gene ontology (GO) classification of differentially expressed genes in the carcinoma and papillary adenocarcinoma tumors into Cellular component, Molecular function and Biological process categories.** Genes that were differently expressed in the two types of tumors (over twofold and at *P *≤ 0.05) were classified to elucidate the cellular subcompartments, functions and processes that account for the differences between phenotypes. Numbers above bars represent the number of genes involved.

A perspective on the cellular components, molecular functions and biological processes involved in the divergent gene expression between phenotypes was obtained by GO Slim classification of the set of 865 features (Figure 2 and Additional file 1, data sheets D-F). A high number of differentially expressed genes were associated with two cellular components: the nucleoplasm and the plasma membrane. In contrast, a relatively low number of these genes were associated with the cytoplasm, most of which were tied to the cytoskeleton. Nucleic-acid binding, catalytic activity and activities linked to maintaining cellular structure were the main molecular functions involving the differentially expressed genes. Differences in the expression of genes involved in signal-transduction activity and transporter activity were also noted. The variance among the resulting biological processes was low, but a markedly higher number of genes were found to be involved in "regulation of gene expression, epigenetic". This definition assembles genes in a relatively wide context that involves cytoplasmic processes that are mitotically or meiotically heritable and do not entail a change in DNA sequence.

### Clustering the differentially expressed genes

**Figure 3:**
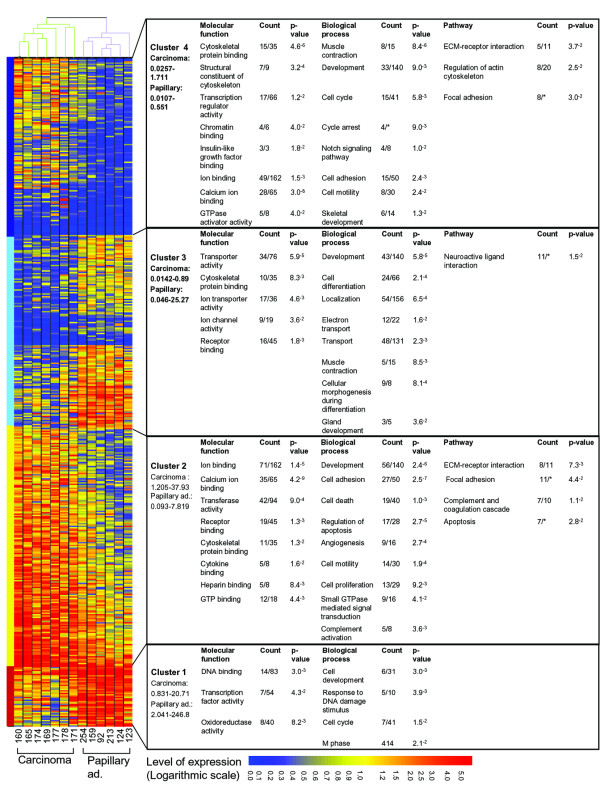
**Unsupervised hierarchical clustering and gene ontology (GO) analysis of genes that are differentially expressed in carcinoma and papillary adenocarcinoma tumors.** Genes with statistically significant (*P *< 0.05) and over twofold differences in their expression levels between the two tumor phenotypes were clustered. In each cluster, significant molecular functions, biological processes and metabolic pathways were calculated using DAVID software. The values below the name of the phenotype in each cluster were determined by "GeneSpring" and indicate relative range of expression compared to the median of gene expression in the intact mammary gland. The intact mammary gland value is 1.00. Counts are the number of genes associated with each definition in a specific cluster, which were related to the number of relevant genes in all clusters. *Total number of relevant genes is not available.

Additional information on the genes that were differentially expressed in the two types of tumors was obtained by applying an unsupervised hierarchical-clustering algorithm. It organizes genes according to the similarity or dissimilarity in expression profile, placing the cases with similar expression profiles together as neighboring rows in the clustergram. Based on normalization of each gene value to the median of gene expression in the intact mammary gland, this analysis assembled the genes into two cluster pairs (Figure 3 and Additional file 1, data sheets G and H). The analysis is Clusters 1 and 3 contained genes that are highly expressed in papillary adenocarcinomas, while clusters 2 and 4 included genes that are preferentially expressed in the carcinoma tumors. Relatively higher levels of gene expression, as compared to the wild-type tissue as well, characterized clusters 1 and 3 relative to clusters 2 and 4, respectively. This distinction allowed further classification of gene expression within each phenotype.

Cluster 1 contained the smallest number of genes: 78 features (72 genes) with relatively high expression levels in the papillary adenocarcinoma compared to the carcinoma tumors or the wild-type gland. These genes mediate molecular functions of nuclear DNA binding, transcription-factor activity and oxidoreductase activity. The main biological processes involve DNA binding, transcription-factor activity and mediating responses to DNA damage. Cluster 3 contained 250 features (241 genes) that were also more highly expressed in the papillary adenocarcinoma, although at a lower level than those listed in cluster 1. In the carcinoma tumors, all of these genes were expressed at lower levels than in the wild-type gland. Contrary to the nuclear activities implicated for most of the genes in cluster 1, the genes in cluster 3 regulate cytoplasmic and membranal activities such as transporter activity, ion-channel activity and cytoskeletal protein binding. Consequently, a wide set of biological processes were affected, including development, cellular morphogenesis and muscle contraction.

Genes with higher expression levels in the carcinoma vs. papillary adenocarcinoma tumors were assembled in clusters 2 and 4. In cluster 2 there were 309 features (270 genes) which also showed high expression levels relative to the wild-type mammary gland. Many of these genes encode binding proteins: ion binding (especially calcium-ion binding), receptor binding, cytoskeletal-protein binding, heparin binding and GTP binding. These proteins assemble to regulate cell adhesion, cell death and apoptosis, as well as cell motility. They maintain cell-cell and cell-extracellular matrix (ECM) interactions, and complement system activity and apoptosis. Cluster 4 included 228 features (203 genes) which were also more highly expressed in the carcinoma, but at relatively lower levels than those in cluster 2. In the papillary adenocarcinoma, these genes were expressed at lower levels that in the wild-type tissue. These genes encode proteins which are involved with the cytoskeleton and calcium-binding activities as well as IGF-I binding and chromatin binding. They complement the biological processes of better ECM-receptor interaction and focal adhesion shown for cluster 2, but are specifically involved in regulating actin-cytoskeleton activities and the Notch pathway.

### Assigning the differentially expressed genes to metabolic pathways

**Figure 4:**
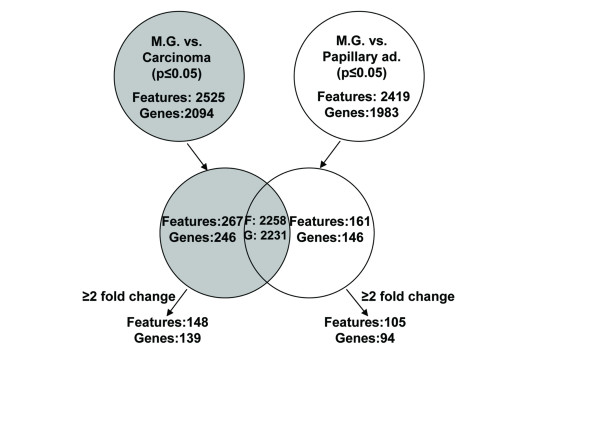
**Comparison of genes with differential expression in tumors vs. wild-type mammary gland.** Genes that are exclusively affected in each phenotype were determined.

To better understand the reasons for the differential expression of genes in the two tumor phenotypes, we distinguished those which were affected by the tumorigenic process in each of the two tumor types (Figure 4). Of the 2,525 or 2,419 features (2,094 or 1,983 genes, respectively) that were differentially expressed in, respectively, the carcinoma or papillary adenocarcinoma tumors as compared to the mammary gland at *P *< 0.05, only 10 and 7%, respectively, were exclusively affected. Fifty-five and 66% of those, respectively, also showed an over twofold difference in their expression levels. We integrated this information in the following metabolic-pathway analysis.

**Figure 5:**
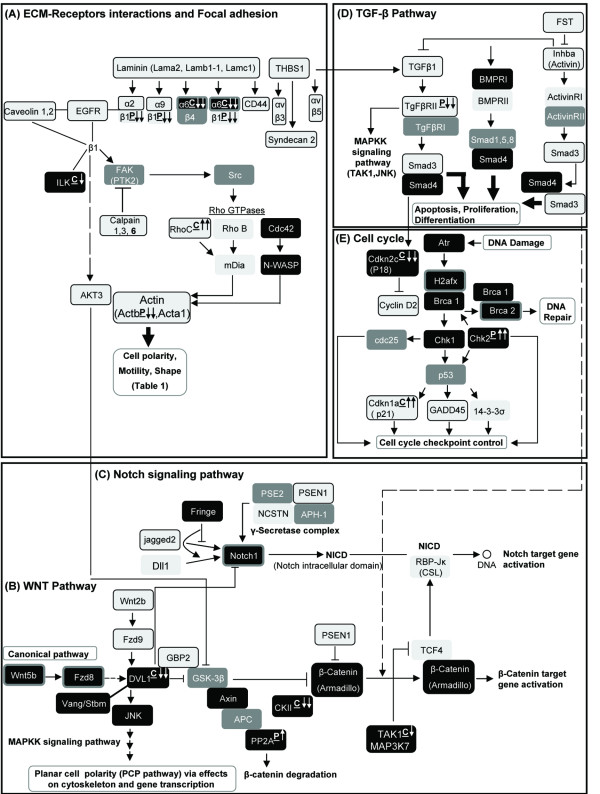
**Genes with differential expression in the carcinoma and papillary adenocarcinoma tumors were assembled into distinct metabolic pathways.** KEGG pathway analysis was performed using DAVID software. Light gray background – significantly (*P *< 0.05) higher expression in carcinoma; gray background – no significant difference; black background – significantly (*P *< 0.05) higher expression in papillary adenocarcinoma; frame around background – over twofold difference. The underlined letters C (carcinoma) or P (papillary adenocarcinoma) which follow the gene name indicate an exclusive effect in one of the two tumor phenotypes compared to the mammary gland. The direction of the arrows indicates the type of change (up- or downregulation). One arrow – statistically significant (*P *< 0.05) difference. Two arrows – statistically significant and over twofold difference.

Genes that were differentially expressed in the two phenotypes were assembled into metabolic pathways (Figure 5 and Additional file 1, data sheet I). A set of genes was identified coding for distinct integrin subunits which were expressed at higher levels in carcinoma than in papillary adenocarcinoma tumors. As αβ-heterodimers, the integrins serve as receptors for ECM components, some of which (laminin and thrombospondin 1 – THBS1) are also highly expressed in the carcinoma tumors (Figure 5A). Thus, integrin α2 or α9 interacts with integrin β1 to form collagen (I, II, IV) receptors. Integrin α2 interacts with integrin β1 to form laminin or THBS1 receptors, and integrin αV interacts with integrin β3 or β5 to form RGD as well as THBS1 receptors [20,21]. All integrins except α6β4 are linked to the actin-based microfilament system, which the integrins also regulate and modulate [20]. The cytoplasmic domain of the β4 subunit is larger, connecting to intermediate filaments instead of to actin [20]. Interestingly, integrin β4 was not affected by the phenotypic deviation and integrin α6 expression was significantly higher in the papillary tumors, mainly due to decreased expression in the carcinoma cells as compared to the wild-type mammary gland. This may imply that the difference in the tumor cells' motility, discussed further on, involves mainly the actin cytoskeleton.

**Figure 6:**
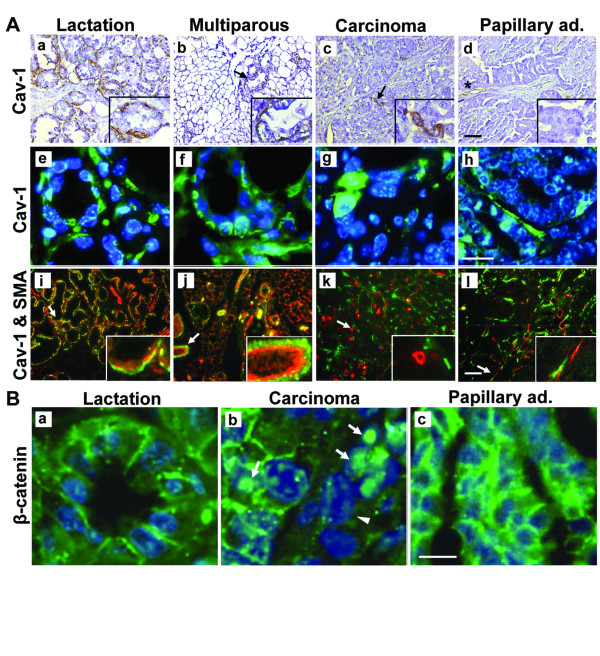
**Cellular and subcellular compartmentalization of caveolin 1 (Cav-1) and β-catenin expression. A. Cav-1 expression. **a-d – Immunohistochemical analysis of Cav-1 expression in mammary gland and tumors. Cav-1 is stained in epithelial cells of the carcinoma tumors vs. the fibrovascular core of the papillary adenocarcinoma tumors. Arrows mark Cav-1 expression in the parenchymatic cells. Asterisk marks Cav-1 expression in fibroblasts. Bar = 20 μm. Inset: 3× magnification. e-h – Immunofluorescent green staining of Cav-1 supports its differential localization in the parenchymatic cells of the carcinoma vs. the fibrovascular core of the papillary adenocarcinoma. Bar = 10 μm. i-l – Immunofluorescence staining of Cav-1 (red) and SMA (green) indicates differential expression in the tumor cells. Bar = 20 μm. Inset: 3× magnification. B. Immunofluorescence localization of β-catenin in mammary gland and tumor cells. Arrows mark nuclear-localized β-catenin in the carcinoma tumors. Arrowhead indicates lack of membranal expression of β-catenin. Bar = 10 μm.

Caveolin (Cav)-1 and Cav-2 are principal structural components of the caveolar microdomain – a subcompartment of the plasma membrane [22]. Cav-1 has been reported to suppress cell transformation and its absence promotes early steps in mammary tumor formation in mice [22]. However, several lines of evidence have suggested that Cav-1 may have oncogenic properties leading to breast cancer [23-26]. Significantly (*P *< 0.05) higher levels of Cav-1 and Cav-2 expression were detected in the carcinoma tumors compared to their papillary adenocarcinoma counterparts (2.8- and 3.8-fold, respectively). This difference resulted from an exclusive increase in Cav-2 expression in the carcinoma tumors compared to the wild type mammary gland which was not found in the papillary adenocarcinomas. For Cav-1, higher levels of expression were detected in the wild type mammary gland compared to both types of tumors. Cav-1 expression has been reported to be confined to the myoepithelial cells of the mouse mammary gland [27]. Their absence in the papillary adenocarcinoma implies other targets for expression. Immunohistochemical and immunofluorescence analyses confirmed Cav-1 expression in basal-like epithelial cells of the lactating gland and possibly in a few myoepithelial cells which are stained by both Cav-1 and smooth muscle actin (SMA) (Figure 6Aa,e,i). In the multiparous gland, Cav-1 expression was augmented in the ductal epithelial cells (Figure 6Ab,f,j), as has also been observed in the breast tissue [26]. The carcinoma tumor sections were characterized by non-overlapping expression of Cav-1 and SMA. Cav-1 was detected in the round red-stained epithelium-like cells, but not in the green-stained SMA ones (Figure. 6Ac,g,k). In contrast, in the papillary adenocarcinoma, Cav-1 staining was limited to fibroblasts and endothelial cells of the fibrovascular core (Figure 6Ad,h,l).

Genes coding for cytoplasmic proteins that are downstream of the integrins and implicated in cell adhesion and motility were generally also more highly expressed in the carcinoma tumors (Figure 5A). Among these encoded proteins are the Rho B and Rho C small GTPases and calpain 1, 3, and especially 6. The calpains are highly conserved intracellular, non-lysosomal, calcium-dependent proteases that prompt disassembly of focal-adhesion structures, leading to the turnover of the integrin-dependent cell-matrix adhesion needed for cell movement [28]. Interestingly, Cdc42 and its downstream N-WASP, which induces filopodia [29], were highly expressed in the papillary adenocarcinomas.

The higher expression of actin in the carcinoma tumors is associated with the comparable expression profile of genes encoding cytoskeletal proteins. These genes are listed in Table 1 and include myosins, tropomyosins, troponin and ADP-ribosylation factors. Genes encoding proteins that promote Ca^++ ^sensitization such as troponin T1 (skeletal slow), calmodulin, cam kinase, caldemon and TNF were also highly expressed in the carcinomas. In contrast, those promoting Ca^++ ^desensitization, such as the MLCPs (Ppm1a, Ppm1g, Pp2r2d, Ppp2r1a, Ppp1 catalytic subunit and Cdc42), were more highly expressed in the papillary adenocarcinoma tumors.

**Table 1:** Genes mediating focal adhesion and cytoskeletal activity that differ significantly (P ≤ 0.05) in their expression levels between carcinoma and papillary adenocarcinoma tumors.

**Systematic name**	**Comparative expression**	**Common name**	**Description**
1448346_at	car > pap	Cfl1	cofilin 1, non-muscle
1417752_at	car > pap	Coro1c	coronin, actin-binding protein 1C
1452651_a_at	car > pap	Myl1	myosin, light polypeptide 1, alkali; atrial, embryonic
1448394_at	car > pap	Myl2	myosin, light polypeptide 2, regulatory, cardiac, slow
1427769_x_at	car > pap	Myl3	myosin, light polypeptide 3, alkali; ventricular, skeletal, slow
1449551_at	car > pap	Myo1c	myosin IC
1450650_at	car > pap	Myo10	myosin X
1422544_at	car > pap	Myo10	myosin X
1423049_a_at	car > pap	Tpm1	tropomyosin 1, alpha
1449577_x_at	car > pap	Tpm2	tropomyosin 2, beta
1425028_a_at	car > pap	Tpm2	tropomyosin 2, beta
1449997_at	car > pap	Tpm3	tropomyosin 3, gamma
1449996_a_at	car > pap	Tpm3	tropomyosin 3, gamma
1450813_a_at	car > pap	Tnni1	troponin I, skeletal, slow 1
1419606_a_at	car > pap	Tnnt1	troponin T1, skeletal, slow
1418884_x_at	car > pap	Tuba1	tubulin, alpha 1
1416311_s_at	car > pap	Tuba7	tubulin, alpha 7
1426427_at	car > pap	Ttll1	tubulin tyrosine ligase-like 1
1424768_at	car > pap	Cald1	caldesmon 1
1417366_s_at	car > pap	Calm1	calmodulin 1
1426098_a_at	car > pap	Cast	calpastatin
1423058_at	car > pap	Capza2	capping protein (actin filament) muscle Z-line, alpha 2
1423057_at	car > pap	Capza2	capping protein (actin filament) muscle Z-line, alpha 2
1434036_at	car > pap	Mtss1	metastasis suppressor 1
1423588_at	car > pap	Arpc4	actin-related protein 2/3 complex, subunit 4
1436722_a_at	car > pap	Actb	actin, beta, cytoplasmic
1427735_a_at	car > pap	Acta1	actin, alpha 1, skeletal muscle
1456473_x_at	car > pap	Arf2	ADP-ribosylation factor 2
1416459_at	car > pap	Arf2	ADP-ribosylation factor 2
1437331_a_at	car > pap	Arf3	ADP-ribosylation factor 3
1423973_a_at	car > pap	Arf3	ADP-ribosylation factor 3
1421789_s_at	car > pap	Arf3	ADP-ribosylation factor 3
1418822_a_at	car > pap	Arf6	ADP-ribosylation factor 6
1424307_at	car > pap	Arhgap1	Rho GTPase-activating protein 1
1426952_at	car > pap	Arhgap18	Rho GTPase-activating protein 18
1417225_at	car > pap	Arl6ip5	ADP-ribosylation factor-like 6 interacting protein 5
1424021_at	car > pap	Arl6ip6	ADP-ribosylation factor-like 6 interacting protein 6
1435559_at	pap > car	BC029719	myosin VI
1422536_at	pap > car	Tnni3	troponin I, cardiac 3
1438608_at	pap > car	Tnni2	troponin I, skeletal, fast 2
1434588_x_at	pap > car	Tbca	tubulin cofactor a
1447964_at	pap > car	Ttl	tubulin tyrosine ligase
1452193_a_at	pap > car	Wasl	Wiskott-Aldrich syndrome-like (human)
1434074_x_at	pap > car	Arf4	ADP-ribosylation factor 4
1431429_a_at	pap > car	Arl4	ADP-ribosylation factor-like 4

The Wingless-type (Wnt) pathway includes genes that are involved in transcription and cell adhesion (Figure 5B). The array analysis displayed a clear-cut differentiation in Wnt-related gene activation in the carcinoma vs. papillary adenocarcinoma tumors. Wnt5b and its Frizzled receptor Fzd8, which activate the non-canonical pathway, were more highly expressed in the papillary adenocarcinoma tumors. Higher expression of downstream Dishevelled-1 (Dvl-1) and JNK, which activate the PCP pathway and control cell movement, were also noted [30]. These changes were associated with higher expression of genes in the canonical pathway that encode proteins of the degradation complex. Those proteins target β-catenin to ubiquitination and proteolysis, rather than translocation into the nucleus. Thus, axin, PP2A and CKII were more highly expressed in the papillary adenocarcinoma, either due to enhanced expression compared to the wild-type mammary gland, or to downregulation of their expression in the carcinoma.

One notable characteristic of the carcinoma tumors was high expression of Wnt2b and Fzd9, which cause distinct activation of the canonical pathway, leading to increased transcription [31]. The lower expression levels of the genes encoding the degradation complex in this pathway could allow better migration of β-catenin into the nucleus where it binds transcription factors, one of which is TCF. Indeed, TCF gene expression was significantly higher in the carcinoma than in the papillary adenocarcinoma tumors, while expression of TAK1, which blocks its nuclear localization and DNA binding [32], was higher in the papillary adenocarcinomas.

Because the array data suggested differential expression of β-catenin in the subcellular compartments of the tumor cells, immunofluorescence analysis of β-catenin was performed (Figure 6B), and nuclei expressing β-catenin were quantitated in five microscopic fields (×40) from sections of five tumors of each type and three wild-type lactating glands. In the lactating gland, β-catenin was located in the nuclei of 40 ± 5.5% of the epithelial cells (Figure 6Ba). A higher number of cells exhibiting visible levels of nuclear β-catenin were observed in the carcinoma tumors (81 ± 2.4% of the cell population, Figure 6Bb). In contrast, the papillary adenocarcinoma sections were almost completely devoid of detectable nuclear β-catenin: it was detected in the nuclei of only 0.3 ± 0.1% of the cells, but was highly expressed in the cytoplasm (Figure 6Bc).

The Notch system plays a predominant role in breast cancer and interacts with other pathways, including the Wnt-signaling pathway [33,34]. Notch signaling is triggered by the binding of one of five different membrane-bound ligands to one of the Notch proteins on neighboring cells. This interaction leads to its proteolitic cleavage and release of the Notch intracellular domain (NICD), which translocates into the nucleus to activate gene transcription. In our analysis, Notch 1 expression was significantly higher in the papillary adenocarcinomas (Figure 5C). However, the expression of its ligands, Jagged 2 and Dll1, was higher in the carcinoma tumors. Interestingly, the papillary adenocarcinoma also exhibited a higher expression level of Fringe – a β-1,3-N-acetylglucosaminyltransferase that modifies Notch receptors and alters their ligand-binding specificity towards the Dll ligands [35,36]. Thus, the papillary adenocarcinoma may preferentially adopt Dll-Notch signaling over that of Jagged. While cytoplasmic Notch 1 is more highly expressed in the papillary adenocarcinoma, the members of the γ-secretase complex, PSN1 and nicastrin (NCSTN), which release its NICD, were highly expressed in the carcinoma tumors. Nuclear activity of the NICD depends on its binding RBP-Jk, which was also highly expressed in the carcinomas.

The TGF-β super-family comprises TGF-βs, bone morphogenetic protein (BMP) and activin. TGF-β or activin bind type II serine/threonine kinase-coupled receptor to further recruit and phosphorylate the type I receptor. In turn, the type I receptor recruits and phosphorylates the R-Smads (for example Smad3) which dissociate and interact with the collaborating Smad, Smad4, to affect gene expression [37,38]. BMP ligands have a higher affinity to the extracellular domain of BMPRI, which then assembles BMPRII. In the current study, TGF-β1 and TGF-βRII were found to be highly expressed in the carcinoma tumors along with the downstream Smad3 (Figure 5D). Activin and its Type I receptor were also highly expressed in these poorly differentiated tumors. In contrast, Smad4, which oligomerizes with Smad3 to form a trimeric protein complex, was more highly expressed in the papillary adenocarcinomas and may enhance the effect of Smad3 in these tumors.

DNA damage or abnormally structured DNA triggers multiple checkpoint pathways that arrest cell-cycle progression [39]. Failure to arrest cell-cycle progression before or at mitosis causes aberrant chromosomal segregation, asymmetric division, aneuploidy and cancers [40]. The mitotic catastrophes influence regulatory protein expression. In the papillary adenocarcinoma tumors, proteins encoded by genes involved in the early steps of the response to DNA damage were more highly expressed than in the carcinomas (Figure 5E). This included the Atr which functions to recognize DNA damage and histone H2afx – a downstream effector involved in DNA repair via chromatin modeling [39]. The papillary adenocarcinoma also exhibited higher expression of BRCA1 and BRCA2, which are involved in DNA repair, Chk1 and Chk2 – highly mobile messengers which are capable of "globally" spreading the DNA-damage-induced signal throughout the nucleus [41], and the cyclin-dependent kinase inhibitor p18(INK4c) [42]. G2/M – a later checkpoint, prevents cells from entering into mitosis via inhibition of Cdc2/cyclin B. Interestingly, three key proteins that regulate this checkpoint are encoded by genes that were highly expressed in the carcinoma tumors: p21, which inhibits Cdc2 directly, 14-3-3σ, which anchors Cdc2 in the cytoplasm where it cannot induce mitosis, and GAdd45, which specifically dissociates Cdc2 from cyclin B1 by co-association with Cdc2 [39,41]. p21 regulates also G1/M, but its involvement also in this later step together with GAdd45 and 14-3-3σ could be indicatory.

**Figure 7:**
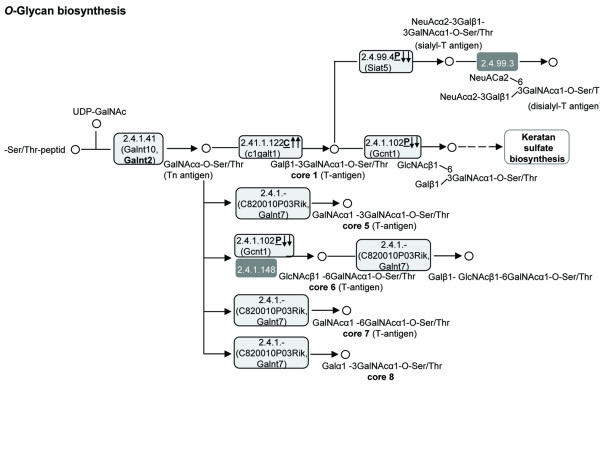
**Higher expression of genes involved in *O*-glycan synthesis in the carcinoma compared to the papillary adenocarcinoma tumors.** KEGG pathway analysis was performed using DAVID software. Light gray background – significantly (*P *< 0.05) higher expression in carcinoma; gray background – no significant difference; frame around background – over twofold difference. The underlined letters C (carcinoma) or P (papillary adenocarcinoma) which follow the gene name indicate the type of tumor in which gene expression is exclusively different from the mammary gland. The direction of the arrows indicates the type of change (up- or downregulation). One arrow – statistically significant (*P *< 0.05) difference. Two arrows – statistically significant and over twofold difference.

Glycosylation is an important post-translational modification of many biologically relevant molecules. A change in the structure of the glycans added to glycoproteins and glycolipids is a common feature of malignancy [43]. *O*-glycosylation is initiated by the transfer of GalNAcα- to threonine or serine residues of the polypeptide backbone, where eight *O*-glycan core structures can then be synthesized [44]. In the current study, the genes encoding the N-acetylgalactosaminyltransferases that are involved in the generation of core structures 1, 5, 6, 7 and 8 were highly expressed in the carcinoma tumors (Figure 7 and Additional file 1, data sheet I). The extension of core 1 to form the disalyl-T antigen is mediated by sialyltransferase (Siat5). The expression of the gene encoding this enzyme was also higher in the carcinoma cells, as were the expressions of Siat7d and Siat9, which mediate sialylated glycoconjugates involved in sphingolipid metabolism (not shown).

### Validation of Affymetrix data by semi-quantitative reverse transcriptase-polymerase chain reaction (RT-PCR)

**Figure 8:**
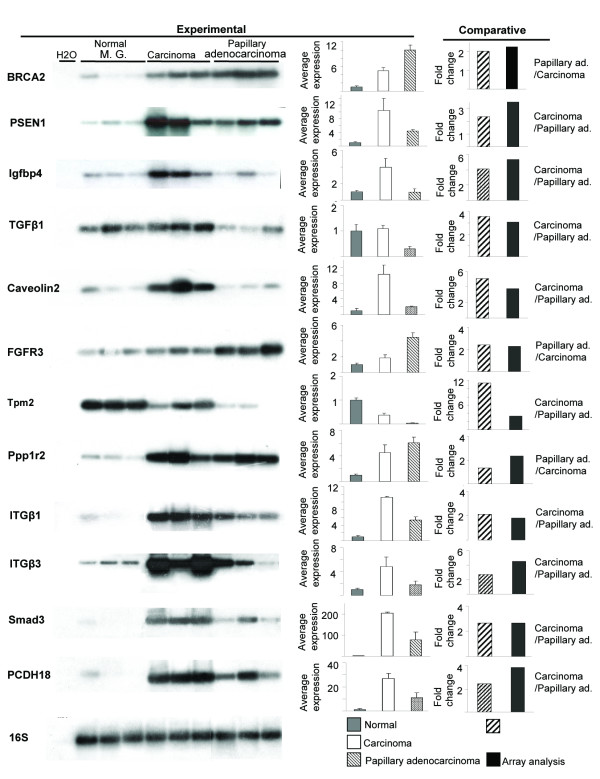
**Validation of the gene-array analysis.** Expression of selected genes in the mammary gland of aged multiparous mice and tumors was analyzed by semi-quantitative RT-PCR. After blotting and hybridization to the relevant probes, signals (on the left) were quantitated and their average intensities ± SEM are presented (Middle panels). The relative expressions of the individual genes in the carcinoma vs. papillary adenocarcinoma were calculated and compared to those obtained from the microarray data (right panels).

To confirm the differences in gene-expression profiles measured by gene-array analysis in the poorly differentiated carcinoma tumors vs. the highly differentiated papillary adenocarcinomas, semi-quantitative RT-PCR analysis was performed (Figure 8). We amplified selected genes' expressions in individual RNA samples extracted from normal mammary glands of aged multiparous females, as well as from tumors of the different phenotypes. Comparable relative expressions of these genes in carcinoma and papillary adenocarcinoma tumors were calculated for the RT-PCR and array analyses.

## Discussion

The number of clearly differentiated phenotypes observed among breast tumors suggests that diversity is an inherent feature of the disease, involving complex but quantifiable parameters. A considerable number of the genes involved in the diversity between the poorly differentiated carcinoma and highly differentiated papillary adenocarcinoma tumors are associated with the nucleoplasm and the plasma membrane. Thus, nuclear dictates expressed by DNA- and RNA-binding activities, and communication processes between cells and their microenvironment, are involved in determining the phenotypical differences between the tumor types. The diversity in genes mediating structural and molecular activities completes the nucleus-cytoskeleton-plasma membrane axis that comprises most of the phenotypical and functional differences between these tumors.

A comparable number of genes were either upregulated or repressed in the carcinoma vs. papillary adenocarcinoma tumors, but only ~10% of these were expressed at higher levels in the papillary adenocarcinoma than in the wild-type gland. Most of these genes encode nuclear proteins that are involved in regulation of the cell cycle and responses to DNA damage and their differential expression may mark the transition from a normal to neoplastic state. As discussed further on, the unique assembly of genes with this type of expression may reflect the significance of genes regulating cell sensitivity to G1 arrest in the papillary adenocarcinoma in determining the diversity among the two types of tumors.

Genes that are relatively more highly expressed in the papillary adenocarcinoma, but at lower levels, encode cytoplasmic proteins, many of which are specific to mammary-gland development and cell differentiation. A representative of these genes, which could potentially serve as a target for therapeutic intervention, is the progesterone receptor (PR) which was expressed at over fourfold higher levels in the papillary adenocarcinoma compared to the carcinoma. The stronger expression of genes involved in transporter activity and ion channels in the papillary adenocarcinoma could reflect the maintenance of cell-cell contact in this loosely adhesive cell population.

The nuclear activity characterizing the highly expressed genes in the papillary adenocarcinoma was not found in the carcinoma tumors. On the other hand, many of the highly expressed genes in the latter tumors encode cytosolic binding proteins: cytoskeletal binding proteins, heparin-binding proteins, GTP-binding proteins, and calcium ion-binding proteins. The stronger expression of these regulatory genes may complement the better interactions of the carcinoma cells with the ECM, as depicted by the higher expression of genes coding for several key integrins. Interestingly, genes with higher expression in the carcinoma tumors, but with a generally lower level of expression, are the backbone of the favored actin-cytoskeleton activity.

Categorization of the genes according to their involvement in particular metabolic functions served to highlight several pathways. Genes involved in ECM-receptor interactions and the focal-adhesion pathway encode proteins of the cell-adhesion sites that are involved in cell detachment and actin-cytoskeleton activities. Most of these genes were more highly expressed in the carcinoma tumors, implicating a higher potential for cell adhesion, membrane protrusion, spreading and migration in these tumors [45]. Since tumors developed in mouse models rarely metastasize [46], the expression profile of these genes may unveil the cryptic motility and metastatic potential of the carcinoma tumors. The stronger expression of N-acetylglucosaminyltransferases and sialyltransferases in the carcinoma tumors potentially augments the anti-adhesive effect of Muc1 and Muc 4, and contributes to tumor progression [44,47].

Cav-1 is also involved in cell adhesion [48] and was expressed at higher levels in the carcinoma tumors than in the papillary adenocarcinomas. Its external expression in the membrane of the basal epithelial cells comprising the neoplastic mass of the carcinoma vs. its internal localization within the papillary fibrovascular core exemplifies the possible involvement of intracellular compartmentalization of gene expression in the determination of tumor phenotypes.

The differential expression of the genes comprising the Wnt pathways led to augmentation of β-catenin in the cytoplasm of the papillary adenocarcinomas as compared to its nuclear translocation in the carcinoma tumors. Accumulation of β-catenin in the cytosol of the mammary tumor cells and formation of cadherin-catenin complexes have been reported to induce cell polarity, survival and more controlled cell proliferation. Those factors are mandatory for assuming a specific shape within the tissue [49] and most likely induce the unique shaping of the papillary adenocarcinoma. High expression of VASP and formin-binding protein in the papillary adenocarcinoma triggers long unbranched filaments termed filopodia, which are frequently observed in cell-cell contacts [50]. When the cadherin-catenin complex is less engaged, such as in the carcinoma, cell death and increased proliferation occur, as well as enhanced migration [49,50].

The Notch pathway regulates the development of postnatal and adult tissues [33]. It has been suggested that its tumor-promoting activity results from its involvement in restricting differentiation towards alternate fates and thereby allowing self-renewal and survival of relatively undifferentiated cells. However, Notch may also exert an anti-proliferative effect via the activation of p21 [51]. Our array analysis indicated distinct expression patterns for genes involved in the Notch pathway in the two types of tumors. The carcinomas exhibited higher expression of the Jagged and Dll1 ligands, as well as of members of the γ-secretase complex which allow their nuclear translocation, and increased Wnt signaling towards cell proliferation [34]. In the papillary adenocarcinoma, however, stronger expression of the Notch-1 receptor was observed and the higher expression of Fringe favors its activation by the Dll rather than Jagged ligand. The role of this pattern of gene expression in specific properties of the papillary adenocarcinoma tumors has yet to be determined. Similarly, the higher expression of Smad4 in the papillary adenocarcinoma tumors as potential compensation for the better expression of members the TGF-β-Smad3 pathway in the carcinomas has to be elucidated.

Differences were also demonstrated in the regulation of cell-cycle activity in the two tumors. Genes involved in G1 arrest and DNA repair were more highly expressed in the papillary adenocarcinoma. In contrast, G2/M arrest, mainly via Cdc2/cyclin B, was favored in the carcinoma tumors. The earlier induction of genes involved in DNA damage, repair and senescence in the papillary adenocarcinoma may account for this tumor's less aggressive phenotype [52].

Our results provide evidence that the poorly differentiated mammary carcinoma and highly differentiated papillary adenocarcinoma represent biologically distinct disease entities. Both types were developed in transgenic mice expressing either the constitutively active Stat5 or its C-terminally truncated counterpart. Manipulation of Stat5 levels and activity during the fertile period in transgenic females may leave a mark on the gene-expression profile in the developing tumors [14]. Together with accumulated microenvironmental and genetic effects, it contributes to the establishment of unique tumor phenotypes. To this end, our analysis suggests that specific differences in gene expression profiles and resulting metabolic pathways contribute to maintaining the carcinoma and papillary adenocarcinoma tumor phenotypes, regardless the transgenic Stat5 variant which was involved in inducing the tumors.

The clinical relevance of our findings is an important issue. To the best of our knowledge, no corresponding comparative study has been performed in breast tumors that would allow direct alignment with our data. However, information suggests that the poorly differentiated DCIS (grade III) is more likely to be associated with invasive human disease than its well-differentiated (grade I) counterparts [12,53-55]. This may be reflected in the higher motility implied by the analysis of the poorly differentiated carcinoma compared to the highly differentiated papillary adenocarcinoma. On an individual gene basis, our data match the results presented in a study determining gene-expression profiles during the transition of breast tumors to invasive stages [56]. The mouse and human lists shared over 30 genes or close family members with higher expression in the high-grade, less differentiated tumors. These include proteosome 26 subunit (PSMD), topoisomerase (TOP), reticulon 4 (RTN4) and CDK, among others. Gene-expression profiles have also been compared in human papillary thyroid carcinomas at different levels of differentiation [57]. In the aggressive, less differentiated groups, a similar increase in the expression of genes encoding the collagen proteins was noted, as well as in those encoding CDC7, TOP2, UBE2 and Rho. In contrast, ESP8 and members of the keratin family were downregulated. The above studies provide lists of genes that were affected by the degree of tumor differentiation. Our data link these genes to a mechanistic base and involve putative pathways that may enable more successful therapeutic intervention.

## Conclusion

Gene-expression profiling in the carcinoma and papillary adenocarcinoma tumors pinpointed distinct molecular functions and major metabolic pathways that are implemented in each tumor to preserve its unique structure and functions. The array data associated the papillary adenocarcinoma with increased expression of genes involved in cell-cell contact and cell-cycle control at an early cell-cycle stage. The carcinoma tumors, on the other hand, displayed increased expression of pathways whose genes are involved in mediating cell-ECM interactions, detachment, motility and spreading. Only a fraction of the genes composing a particular pathway were affected. This suggests a dominant role for their encoded proteins in determining specific tumor properties at this basic gene-expression level. Additional proteomic analysis is expected to complement this study by linking a catalytic activity, mediated by post-transcriptional modifications, to the differential expression of these genes.

## Abbreviations

GO: gene ontology. ECM: extracellular matrix. H&E: hematoxylin & eosin. RT-PCR: reverse-transcription PCR. Stat: signal transducer and activator of transcription. STAT5: transgenic Stat5.

## Competing interests

These authors declare that they have no competing interests.

## Authors' contributions

TE designed and performed the research and also helped in writing the manuscript. IB designed the research and wrote the manuscript.

## Pre-publication history

The pre-publication history for this paper can be accessed here:



## Supplementary Material

Additional file 1 **Sheet A. **List of primers used to amplify coding regions of the following listed genes. Sheet B. List of features with significantly (*P *≤ 0.05) different levels of expression in the carcinoma vs. papillary adenocarcinoma tumors. Sheet C. List of features with significant (*P *≤ 0.05) and an over twofold difference in expression in the carcinoma vs. papillary adenocarcinoma tumors. Sheet D. GO classification: List of genes comprising the cellular component analysis. Sheet E. GO classification: List of genes comprising the molecular function analysis. Sheet F. GO classification: List of genes comprising the biological process analysis. Sheet G. List of genes with significant (*P *≤ 0.05) and an over twofold difference in expression in carcinoma vs. papillary adenocarcinoma tumors comprising the four clusters. Sheet H. List of genes included in the DAVID GO analysis of the four clusters. Sheet I. List of genes with differential expression in the carcinoma and papillary adenocarcinoma tumors that were assembled into the distinct metabolic pathways. Click here for file
